# The peroxisomal protein import machinery displays a preference for monomeric substrates

**DOI:** 10.1098/rsob.140236

**Published:** 2015-04-08

**Authors:** Marta O. Freitas, Tânia Francisco, Tony A. Rodrigues, Celien Lismont, Pedro Domingues, Manuel P. Pinto, Cláudia P. Grou, Marc Fransen, Jorge E. Azevedo

**Affiliations:** 1 Organelle Biogenesis and Function Group, Instituto de Biologia Celular e Molecular (IBMC), Universidade do Porto, Porto, Portugal; 2 Instituto de Ciências Biomédicas Abel Salazar (ICBAS), Universidade do Porto, Porto, Portugal; 3 Departamento de Química, Universidade de Aveiro, Aveiro, Portugal; 4 Departement Cellulaire en Moleculaire Geneeskunde, KU Leuven–Universiteit Leuven, Leuven, Belgium

**Keywords:** peroxisomes, PEX5, docking/translocation machinery, acyl-CoA oxidase, urate oxidase, protein import

## Abstract

Peroxisomal matrix proteins are synthesized on cytosolic ribosomes and transported by the shuttling receptor PEX5 to the peroxisomal membrane docking/translocation machinery, where they are translocated into the organelle matrix. Under certain experimental conditions this protein import machinery has the remarkable capacity to accept already oligomerized proteins, a property that has heavily influenced current models on the mechanism of peroxisomal protein import. However, whether or not oligomeric proteins are really the best and most frequent clients of this machinery remain unclear. In this work, we present three lines of evidence suggesting that the peroxisomal import machinery displays a preference for monomeric proteins. First, in agreement with previous findings on catalase, we show that PEX5 binds newly synthesized (monomeric) acyl-CoA oxidase 1 (ACOX1) and urate oxidase (UOX), potently inhibiting their oligomerization. Second, *in vitro* import experiments suggest that monomeric ACOX1 and UOX are better peroxisomal import substrates than the corresponding oligomeric forms. Finally, we provide data strongly suggesting that although ACOX1 lacking a peroxisomal targeting signal can be imported into peroxisomes when co-expressed with ACOX1 containing its targeting signal, this import pathway is inefficient.

## 2. Introduction

Peroxisomes are single membrane-bounded organelles that participate in many biochemical pathways [1–4]. In mammals, they have a relatively simple protein repertoire, harbouring about 50 enzymes in their matrix [5,6]. All these proteins are synthesized on cytosolic ribosomes and post-translationally targeted to the organelle matrix [7]. Their correct sorting relies on one of two peroxisomal targeting signals (PTS): the PTS type 1 (PTS1), a C-terminal peptide generally ending with the sequence Ser-Lys-Leu, found in the vast majority of peroxisomal matrix proteins [8,9]; and the PTS2, a degenerate nonapeptide present at the N-termini of just a few proteins [10–12].

According to current models [13–16], newly synthesized peroxisomal matrix proteins are recognized by the shuttling receptor PEX5 while still in the cytosol [17]. PTS1 proteins bind directly to PEX5, whereas PTS2 proteins require the ancillary protein PEX7 to interact with PEX5 [8,12,18–21]. Following this recognition event, the PEX5(.PEX7)–cargo protein complex interacts with the docking/translocation machinery (DTM) [22–24], a multisubunit transmembrane protein complex of the peroxisome [25,26]. This interaction ultimately results in the insertion of PEX5 into the DTM with the concomitant translocation of the cargo protein across the organelle membrane [23,24,27,28]. Remarkably, none of these steps requires cytosolic nucleoside triphosphates [23,24,28,29], a property that led to the proposal that the driving force for the cargo translocation process resides on the strong protein–protein interactions that are established between PEX5 on one side and components of the DTM on the other [29,30]. After cargo translocation, PEX5 is extracted from the DTM [23,24,28], a process that requires its monoubiquitination at a conserved cysteine residue [31,32] and the ATP-dependent action of the mechano-enzymes PEX1 and PEX6 [29,33,34]. Once in the cytosol, monoubiquitinated PEX5 is deubiquitinated, probably by a combination of enzymatic and non-enzymatic mechanisms, thus resetting the peroxisomal import machinery (PIM) [35–37].

Although our knowledge on the general properties of the PIM is fairly detailed, there are still many fundamental aspects of this protein import pathway that remain ill-defined. An important one regards the structure of the cargo proteins accepted by the PIM. It is a widely accepted fact that peroxisomes can import already oligomerized proteins. The data supporting this idea are abundant and include (i) several studies showing that when two interacting proteins are expressed in the same cell, the presence of a single PTS in one of those proteins is sufficient to ensure targeting of at least a fraction of the other protein to the peroxisome [38–44] and (ii) pulse–chase analyses on yeasts suggesting that two peroxisomal matrix enzymes oligomerize in the cytosol prior to import [45,46]. Collectively, these data led to the generalization that most peroxisomal proteins oligomerize in the cytosol before import, a concept that can be found in many reviews and even in academic textbooks [15,47–51]. However, it should be noted that all of the above cited studies focused on proteins that were overexpressed, either through the use of recombinant genes having strong promoters or, in the case of yeasts/fungi, by simply growing these organisms in special media that induce a dramatic proliferation of peroxisomes. Such experimental conditions can potentially lead to the titration of the PIM (e.g. PEX5 and/or PEX7) and thus to the premature oligomerization of those proteins in the cytosol. Naturally, this caveat does not affect the main conclusion of all those studies, namely that the PIM, in contrast to the protein import machineries of mitochondria and endoplasmic reticulum, has the capacity to accept bulky/already folded clients [39,40]. However, in the absence of additional data, it remains unclear how frequent and efficient the import of already oligomerized proteins into peroxisomes is, an uncertainty that limits our understanding on the mechanism of the PIM (see below).

The uncertainty regarding the concept that most peroxisomal proteins are imported as oligomers is also fed by a number of previous findings. For instance, pulse–chase analyses have shown that rat liver catalase (a homo-tetrameric enzyme in its native state) and *Candida boidinii* alcohol oxidase (an octameric enzyme) arrive at the peroxisome still as monomers [52,53]. Also, some data suggesting that plant monomeric isocitrate lyase (a homo-tetrameric enzyme in its native state) is a better import substrate than the already tetrameric enzyme have been provided [54]. In line with these findings it was subsequently reported that (monomeric) serum albumin containing a PTS is also imported into peroxisomes, clearly showing that cargo proteins do not have to be oligomers in order to be accepted by the PIM [55]. Finally, there are at least three peroxisomal matrix proteins that no longer bind PEX5 upon oligomerization. These are alcohol oxidase from *Hansenula polymorpha* and mammalian carbonyl reductase and epoxide hydrolase [56–58]. Seemingly, at least in these cases, the proteins have to remain monomeric in order to be imported into peroxisomes.

Determining the type of substrate preferred by the PIM is of major importance to understand its mechanism. If we assume that almost all oligomeric peroxisomal proteins oligomerize in the cytosol prior to import, then import of oligomeric cargoes becomes the rule for protein translocation across the peroxisomal membrane, because most peroxisomal matrix proteins are indeed homo-oligomers [48]. This is the scenario behind some previous models proposing that cargoes (containing multiple PTSs due to their oligomeric nature) are presented to the DTM by multiple molecules of PEX5 [48,49]. If, instead, we assume that under normal physiological conditions newly synthesized matrix proteins are kept in a monomeric near-native conformation until they arrive at the organelle matrix, then a model in which a single PEX5 molecule delivers a single cargo to the DTM is more likely [59,60]. The outcomes of each of these assumptions to the cargo protein translocation step are quite different because, as stated above, all the available data suggest that cargo proteins are translocated across the organelle membrane by PEX5 itself, when the receptor becomes inserted into the DTM. Thus, the first scenario would predict that each oligomeric cargo is translocated by several PEX5 molecules (see [15] for a mechanism of this type), whereas in the second scenario a single PEX5 molecule would suffice [59].

Previously, we found that PEX5 at physiological concentrations binds monomeric catalase, potently blocking its tetramerization [61]. This property, together with the fact that there is sufficient PEX5 in rat hepatocyte cytosol to bind all newly synthesized peroxisomal matrix proteins, led us to hypothesize that PEX5, in addition to its role as a receptor and translocator, is also a chaperone/holdase, binding newly synthesized monomeric proteins in the cytosol and inhibiting premature or incorrect interactions [61]. In this work, we have characterized the import pathway of acyl-CoA oxidase 1 (ACOX1; a homo-dimeric protein in its native state [62]) and urate oxidase (UOX; a homo-tetramer [63]), two peroxisomal matrix proteins which together with catalase comprise one-third of the total protein molecules found in mouse/rat liver peroxisomal matrix [62,64]. We found that PEX5 also binds the monomeric version of these proteins, blocking their homo-oligomerization. Importantly, peroxisomal import assays suggest that the monomeric versions of ACOX1 and UOX are much better substrates for the PIM than the corresponding homo-oligomeric versions. Altogether, these results suggest that import of monomeric proteins into the peroxisome is not a phenomenon restricted to a few particular clients. Rather, at the very least, our data raise the possibility that many of the protein translocation events occurring at the PIM involve monomeric cargoes.

## 3. Results

### 3.1. PEX5 inhibits dimerization of newly synthesized acyl-CoA oxidase 1

We have recently shown that a rabbit reticulocyte lysate-based *in vitro* translation system can be used to prepare monomeric and tetrameric versions of catalase. The amount of each of these species in translation reactions is time-dependent: synthesis reactions performed for a short period of time yielded essentially monomeric catalase; longer incubations led to the conversion of a fraction of the monomeric protein into tetrameric catalase, a process that was strongly inhibited by PEX5 [61]. Here, we determined whether the same experimental strategy could be applied to other oligomeric peroxisomal matrix proteins. The aim was twofold: (i) to characterize the effect of PEX5 on their oligomerization process and (ii) to obtain monomeric and oligomeric versions of these proteins so that their *in vitro* peroxisomal import competences could be compared (note that all our attempts to import monomeric or tetrameric catalase into rat/mouse liver peroxisomes have failed thus far, probably because the PEX5–catalase interaction is too transient, and therefore too sensitive to the competition exerted by endogenous (liver) soluble PTS1 proteins present in these *in vitro* assays; T Francisco, JE Azevedo, unpublished observations, see also [23]). We focused mainly on ACOX1, but some experiments were repeated with another peroxisomal matrix protein (see later). Native ACOX1 comprises two identical 74 kDa subunits, each of which is partially and slowly cleaved in the peroxisomal matrix *in vivo* into an N-terminal domain of 53 kDa and a C-terminal 21 kDa polypeptide [65–67].

**Figure 1.**
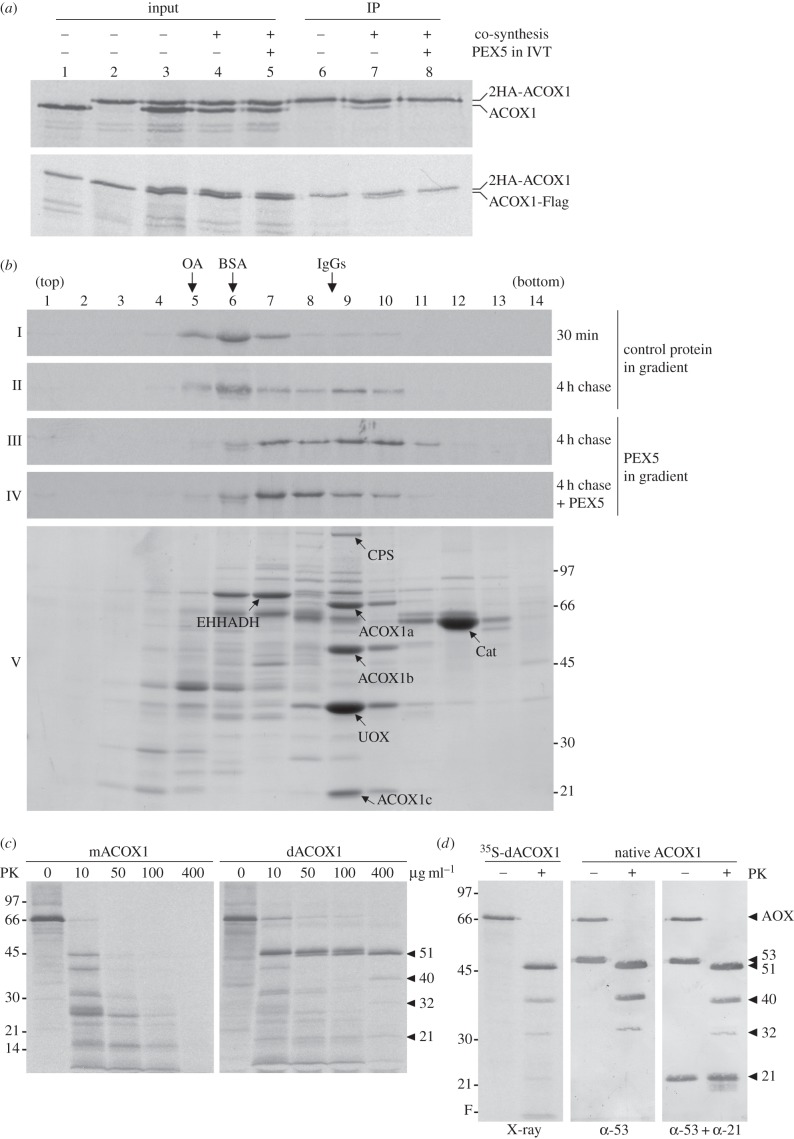
Newly synthesized ACOX1 dimerizes *in vitro*, a process inhibited by PEX5. (*a*) ACOX1 dimerizes *in vitro. Upper panel*: ACOX1 and HA-tagged ACOX1 (2HA-ACOX1) were synthesized individually (lanes 1 and 2) or co-synthesized in the absence (−) or presence (+) of 1 µM recombinant PEX5 (lanes 4 and 5, respectively) for 30 min and subjected to a 4 h chase. A mixture of the two proteins synthesized individually (lane 3) and the co-synthesis reactions (lanes 4 and 5) were subjected to immunoprecipitation (IP) using anti-HA antibody agarose beads (lanes 6–8, respectively). Note that all samples were made chemically identical before immunoprecipitation by adding recombinant PEX5. *Lower panel*: An identical experiment was performed using 2HA-ACOX1 and ACOX1-Flag. IVT, *in vitro* transcription/translation. (*b*) Sedimentation behaviour of *in vitro* synthesized ACOX1. ACOX1 synthesized for 30 min (panel I), and ACOX1 synthesized for 30 min and chased for 4 h in the absence (panels II and III) or presence of 1 µM PEX5 (panel IV) were loaded onto the top of sucrose gradients supplemented with 1 µM of either PEX5 (panels III and IV) or a control protein (panels I and II). After centrifugation, fractions were collected from the bottom of the gradients and subjected to SDS-PAGE/autoradiography. Ovalbumin (OA), bovine serum albumin (BSA) and immunoglobulins (IgGs) were used as sedimentation coefficient standards. Peroxisomal matrix proteins from mouse liver were also subjected to this analysis. A Coomassie-stained gel is shown (panel V). Carbamoyl phosphate synthetase (CPS), 2-enoyl-CoA hydratase/3-hydroxyacyl-CoA dehydrogenase (EHHADH), acyl-CoA oxidase I subunits a, b and c (ACOX1a, ACOX1b, ACOX1c, respectively) and catalase (Cat) were identified by nano-HPLC-MALDI-MS/MS (data not shown). (*c*) Monomeric and dimeric ACOX1 present different susceptibilities to proteinase K. ^35^S-mACOX1 and ^35^S-dACOX1 isolated from a sucrose gradient were treated with increasing concentrations of proteinase K (PK) for 40 min on ice. After protease inactivation, samples were analysed by SDS-PAGE/autoradiography. Numbers to the left indicate the molecular weights of protein standards. Arrow heads indicate proteolysis fragments of ACOX1 (see main text). (*d*) Dimeric ^35^S-ACOX1 and native/peroxisomal ACOX1 display the same proteolysis profile. ^35^S-dACOX1 isolated from a sucrose gradient and native ACOX1 (from mouse liver purified peroxisomes) were subjected to protease treatment in the presence of Triton X-100 and subjected to SDS-PAGE/autoradiography (left panel) or western blotting using antibodies directed to the 53-kDa ACOX1 polypeptide (central panel). The same blot was reprobed with an antibody directed to the 21-kDa polypeptide of ACOX1 (right panel). F, front of the gel.

We first used an immunoprecipitation assay to assess whether ACOX1 can interact with itself in the *in vitro* translation system. As shown in figure 1*a*, upper panel, when ACOX1 and an epitope-tagged version of it containing two haemagglutinin (HA) sequences at the N-terminus (2HA-ACOX1) were co-synthesized *in vitro* for 30 min, chased in the presence of cycloheximide for 4 h, and subjected to immunoprecipitation using an anti-HA antibody, a significant amount of ACOX1 was co-immunoprecipitated with 2HA-ACOX1 (lane 7). No ACOX1 was recovered in immunoprecipitates when the two proteins were synthesized separately for 30 min and mixed just before immunoprecipitation, or when the co-synthesis and chase incubations were performed in the presence of recombinant PEX5 (figure 1*a*, upper panel, lanes 6 and 8, respectively). For reasons that will become apparent below, we used the same strategy to determine whether 2HA-ACOX1 can interact with an ACOX1 species possessing a Flag epitope at its C-terminus (ACOX1-Flag). The results shown in figure 1*a*, lower panel, suggest that this is indeed the case.

In another approach, we used sucrose gradient centrifugation to characterize the sedimentation behaviour of *in vitro* synthesized ACOX1. As shown in figure 1*b*, ACOX1 synthesized for just 30 min sedimented as a globular monomeric protein, together with albumin, a 67 kDa protein (panel I, lane 6). We refer to this species as monomeric ACOX1 (mACOX1). When mACOX1 was chased for 4 h in the presence of cycloheximide, a second ACOX1 population was detected in these gradients (figure 1*b*, panel II, lane 9). Its sedimentation behaviour is identical to the one of native/dimeric mouse liver ACOX1 (figure 1*b*, panel V, bands marked ACOX1a/b/c in lane 9). This species is hereafter referred to as dimeric ACOX1 (dACOX1). Centrifugation of the two ^35^S-labelled ACOX1 populations in the presence of recombinant PEX5 resulted in a slight increase in their sedimentation coefficients (figure 1*b*, panel III), suggesting that both species interact with PEX5. In agreement with the data shown in figure 1*a*, less dACOX1 was generated in translation reactions chased for 4 h in the presence of PEX5. In this case, the major ACOX1 population was detected in fractions 7–8, a behaviour identical to the one observed for mACOX1 in the sucrose gradient containing PEX5 (figure 1*b*, compare panels III and IV).

To further characterize the two ACOX1 populations detected in these experiments their resistance to proteolysis was assessed. As shown in figure 1*c* (left panel) ^35^S-mACOX1 is quite sensitive to proteinase K. By contrast, ^35^S-dACOX1 is cleaved by the protease into a major 51 kDa fragment plus three fragments of 40, 32 and 21 kDa (figure 1*c*, right panel). Importantly, an almost identical proteolysis pattern was obtained for native mouse liver dimeric ACOX1, as assessed by western blotting using antibodies directed to the 53 kDa and 21 kDa polypeptides (figure 1*d*). The only difference between the ^35^S-dACOX1 species and endogenous mouse dACOX1 after protease treatment resides in the relative intensity of the 21 kDa fragment which is barely detectable in the ^35^S-labelled protein. The fact that this domain of ACOX1 contains only one of the 19 methionines present in full-length ACOX1 justifies this difference.

In summary, the experiments described above suggest the following: (i) ACOX1 synthesized *in vitro* for a short period of time is a globular monomeric protease-sensitive protein; (ii) further incubation of *in vitro* synthesized mACOX1 results in its partial conversion into dACOX1; and (iii) PEX5 inhibits ACOX1 dimerization.

### 3.2. Peroxisomal *in vitro* import efficiencies of mACOX1 and dACOX1

**Figure 2.**
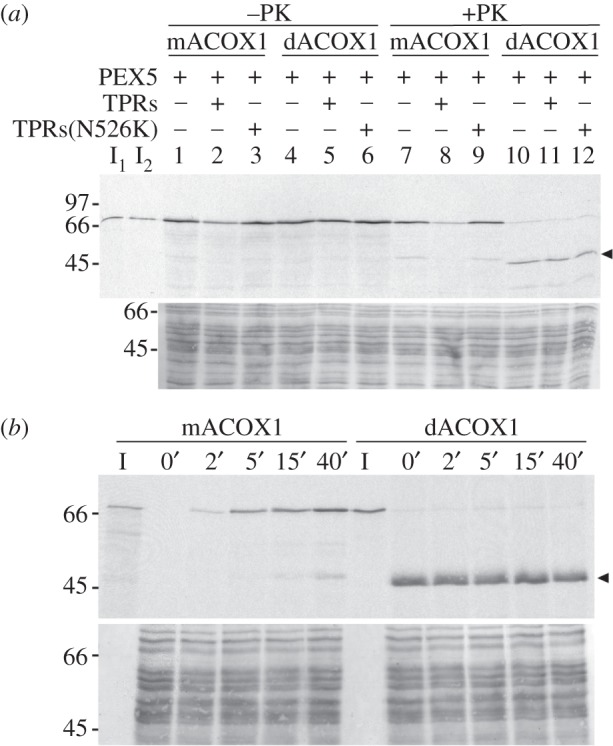
mACOX1 is a better peroxisomal import substrate than dACOX1. (*a*) ^35^S-mACOX1 and ^35^S-dACOX1 isolated from a sucrose gradient were subjected to *in vitro* import reactions in the presence (+) or absence (−) of the indicated recombinant proteins. After incubation, one-half of each sample was treated with proteinase K, as indicated. The organelles were then isolated and analysed by SDS-PAGE/autoradiography. The autoradiograph (upper panel) and the corresponding Ponceau S-stained membrane (lower panel) are shown. I_1_, I_2_—5% of ^35^S-mACOX1 and ^35^S-dACOX1, respectively, used in the assays. The arrow head indicates the 51 kDa protease-resistant fragment of ^35^S-dACOX1. (*b*) Import kinetic analyses of ^35^S-mACOX1 and ^35^S-dACOX1. The two import reactions (each containing 2.5 mg of PNS) were performed in the presence of recombinant PEX5. Aliquots of each import reaction (containing 500 µg of PNS) were withdrawn at the indicated time points, treated with proteinase K and analysed as above. Note that the amount of ^35^S-dACOX1 used in this experiment was approximately twofold that of ^35^S-mACOX1 to obtain similar substrate concentrations. Lanes I—5% of the radiolabelled proteins present in each aliquot.

We next tested the peroxisomal import efficiencies of ^35^S-mACOX1 and ^35^S-dACOX1 using an established *in vitro* import system. For this purpose mACOX1 and dACOX1 obtained from a sucrose gradient as the one presented in figure 1*b* (panel II) were incubated with a rat liver post-nuclear supernatant (PNS) in import buffer supplemented with 1.5 nM recombinant PEX5 (see Material and methods for details). Two control import reactions were included in these experiments. In the first, the import buffer contained also 300 nM of a recombinant protein comprising the C-terminal half of PEX5 (hereafter referred to as TPRs). This truncated PEX5 protein retains the capacity to interact with PTS1-containing proteins but lacks peroxisomal targeting information [68]. Thus, if the concentration of TPRs is much larger than the concentration of PEX5 in the assays then import of ^35^S-labelled ACOX1 should be strongly inhibited. The second control reaction received 300 nM of an inactive version of TPRs (TPRs(N526K)), a protein containing a single missense mutation (N526K) that abolishes its PTS1-binding capacity [18,69]. This recombinant protein should not inhibit PEX5-dependent import of ACOX1. At the end of the incubation, import reactions were treated or not with a large amount of proteinase K and the organelles were isolated by centrifugation and analysed by SDS-PAGE/autoradiography. The results of this experiment are shown in figure 2*a*. Approximately 50% of mACOX1 sedimenting with the organelles acquired a protease-resistant status (compare lanes 1 and 7). An identical result was obtained in the reaction supplemented with TPRs(N526K) (compare lanes 3 and 9), as expected. By contrast, the amount of protease-protected mACOX1 in the reaction supplemented with TPRs was strongly diminished (compare lanes 7 and 8). A different result was obtained for dACOX1. Indeed, although a significant fraction of this protein sedimented with the organelles (lanes 4–6), the vast majority of it remained accessible to the protease (lanes 10–12), indicating that it was not translocated across a membrane. Note that a very small amount of intact dACOX1 is detected in these samples. However, this material is unresponsive to recombinant TPRs (compare lane 11 with lanes 10 and 12) and, therefore, it does not represent authentic imported protein. In agreement with this interpretation, the amount of uncleaved dACOX1 in import assays does not increase over time, in contrast to mACOX1 import (figure 2*b*).

Taken together, the results of these *in vitro* import experiments, although of qualitative nature, strongly indicate that mACOX1 is a far better substrate for the PIM than dACOX1.

### 3.3. Urate oxidase behaves similarly to ACOX1 in the *in vitro* homo-oligomerization and import assays

**Figure 3.**
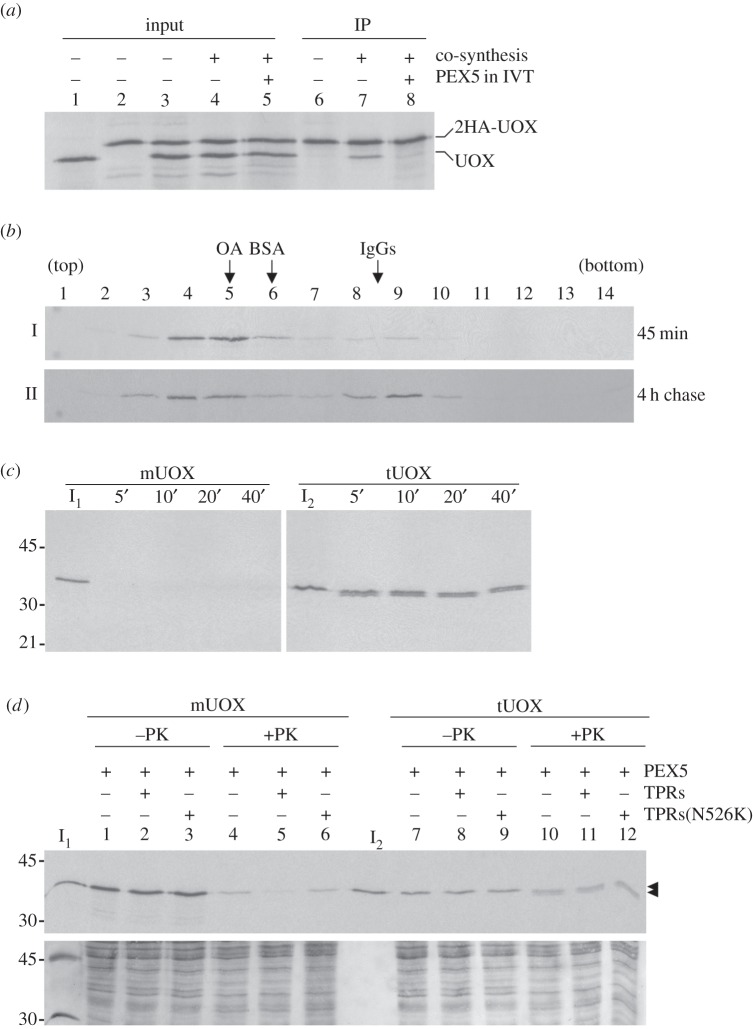
Behaviour of UOX in *in vitro* homo-oligomerization and import assays. (*a*) *In vitro* synthesized UOX oligomerizes in a process inhibited by PEX5. UOX and HA-tagged UOX (2HA-UOX) were synthesized individually (lanes 1 and 2) or co-synthesized in the absence or presence of 1 µM recombinant PEX5 (lanes 4 and 5, respectively) for 45 min, and subjected to a 4 h chase. A mixture of the two proteins synthesized individually (lane 3) and the co-synthesis reactions (lanes 4 and 5) were subjected to immunoprecipitation (IP) using anti-HA antibody agarose beads (lanes 6–8, respectively) and analysed as described in figure 1*a*. IVT, *in vitro* transcription/translation. (*b*) Sedimentation analyses of *in vitro* synthesized UOX. Radiolabelled UOX synthesized for 45 min (panel I) or UOX synthesized for 45 min and chased for 4 h (panel II) were subjected to sucrose gradient centrifugation analyses. The sedimentation positions of ovalbumin (OA), bovine serum albumin (BSA) and immunoglobulins (IgGs) are also shown. (*c*) Monomeric and tetrameric UOX display different susceptibilities to protease treatment. ^35^S-mUOX and ^35^S-tUOX isolated from a sucrose gradient were subjected to proteinase K (PK) treatment (400 µg ml^−1^, final concentration) and aliquots were withdrawn at the indicated time points. Samples were processed as described in figure 1*c*. (*d*) mUOX is a better substrate for the peroxisomal protein import machinery than tUOX. ^35^S-mUOX and ^35^S-tUOX isolated from a sucrose gradient were subjected to *in vitro* import assays in the presence (+) or absence (−) of the indicated recombinant proteins. After incubation, one-half of each sample was treated with proteinase K, as indicated. Isolated organelles were processed and analysed by SDS-PAGE/autoradiography. The autoradiograph (upper panel) and the corresponding Ponceau S-stained membrane (lower panel) are shown. I_1_, I_2_—5% of ^35^S-mUOX and ^35^S-tUOX, respectively, used in the assays. The arrow heads indicate protease-resistant fragments of ^35^S-tUOX.

Aiming at extending the findings obtained with ACOX1 to another peroxisomal protein, we tested UOX in some of the assays described above. UOX, an abundant protein comprising 15% of the total protein molecules found in rat/mouse liver peroxisomes, is a homo-tetramer of 35 kDa subunits in its native state [63,64]. We first asked whether UOX can homo-oligomerize in the rabbit reticulocyte lysate and, if so, whether this process is inhibited by PEX5. The strategy used was exactly the one described above for ACOX1 (figure 1*a*). As shown in figure 3*a*, untagged UOX was co-immunoprecipitated with 2HA-UOX only when the two proteins were co-synthesized in the absence of PEX5 (lane 7). Thus, PEX5 blocks UOX oligomerization.

^35^S-UOX synthesized for just 45 min and ^35^S-UOX subjected to a 4-h chase incubation were also analysed by sucrose gradient centrifugation. As shown in figure 3*b*, the first protein (hereafter referred to as monomeric UOX; mUOX) sedimented slightly above ovalbumin, a 45 kDa monomeric globular protein (panel I, lanes 4 and 5), whereas a fraction of the protein that was allowed to oligomerize (referred to as tetrameric UOX; tUOX) sedimented as authentic native/tetrameric mouse liver UOX (panel II, lane 9; compare with panel V in figure 1*b*). The two species of UOX display quite different behaviours upon proteinase K treatment: mUOX is readily degraded by the protease, whereas tUOX is largely resistant yielding a diffuse doublet that runs slightly below undigested UOX upon SDS-PAGE (figure 3*c*, left and right panels, respectively).

Finally, ^35^S-mUOX and ^35^S-tUOX obtained from a sucrose gradient were tested in *in vitro* import assays. The criteria to define *bona fide* peroxisomal import were the ones used above for ACOX1, i.e. acquisition of a protease-protected, organelle-associated status in a TPRs-inhibitable manner. As shown in figure 3*d*, a small amount of mUOX was imported into peroxisomes (cf. lanes 4 and 6 with 5). By contrast, we were unable to detect specific peroxisomal import of tUOX. Indeed, the radiolabelled protein appearing in the organelle pellets is insensitive to the presence of TPRs in the assays (cf. lanes 10 and 11) and runs as a diffuse doublet upon SDS-PAGE, indicating that it is protease-accessible.

### 3.4. ACOX1 lacking peroxisomal targeting information can be imported into peroxisomes piggybacked with ACOX1 containing the PTS1, but this pathway is inefficient

**Figure 4.**
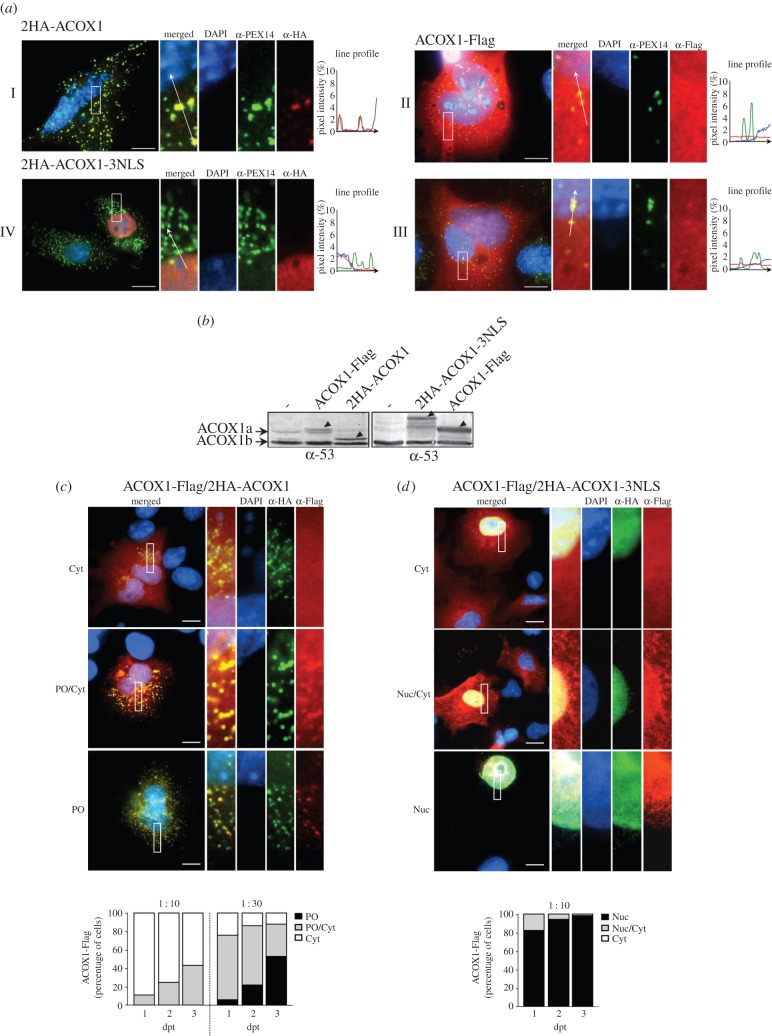
ACOX1 lacking a peroxisomal targeting signal is inefficiently imported into peroxisomes when co-expressed with ACOX1 containing a PTS1. (*a*) COS-7 cells were transfected with plasmids encoding HA-tagged ACOX1 (2HA-ACOX1; panel I), a C-terminally Flag-tagged ACOX1 (ACOX1-Flag; panels II and III), or a HA-tagged ACOX1 containing a nuclear targeting sequence (2HA-ACOX1–3NLS; panel IV). Two days post-transfection the cells were fixed, counterstained with DAPI, and processed for immunofluorescence using an anti-PEX14 antibody (to label peroxisomes) and an anti-HA (panels I and IV) or anti-Flag antibody (panels II and III). Profile plots of fluorescence intensity (in percentage of pixel intensity) along the white arrows shown in the merged panels are also provided: blue line, DAPI staining; green line, anti-PEX14 fluorescence; red line, anti-HA or anti-Flag fluorescence. Scale bar, 10 µm. (*b*) Expression levels of tagged ACOX1 proteins in COS-7 cells. Untransfected cells (lanes ‘—’) or cells transfected with individual plasmids encoding ACOX1-Flag, 2HA-ACOX1 or 2HA-ACOX1–3NLS were analysed by western blot using an antibody against the 53 kDa polypeptide of ACOX1. The arrow heads indicate the tagged ACOX1 proteins. (*c*) COS-7 cells were transfected with mixtures of the plasmids encoding ACOX1-Flag and 2HA-ACOX1 at ratios of 1 : 10 and 1 : 30. Note that, as the total amount of plasmid in each of these mixtures was adjusted to 1 µg, ‘1’ corresponds to 10% and 3.3%, respectively, of the amount of plasmid DNA used in (*a*) and (*b*). The subcellular localization of each protein was then analysed by immunofluorescence 1, 2 and 3 days post-transfection (dpt). Cells in which ACOX1-Flag displays an exclusive cytosolic localization (Cyt; see upper panel for a representative example), a dual peroxisomal/cytosolic localization (PO/Cyt; middle panel), or an exclusive peroxisomal localization (PO; lower panel) were counted and expressed as percentage of ACOX1-Flag-expressing cells in the bar graph. (*d*) Exactly the same co-transfection strategy was used with plasmids encoding 2HA-ACOX1–3NLS and ACOX1-Flag. Cells in which ACOX1-Flag displays an exclusive cytosolic localization (Cyt; see upper panel for a representative example), a dual nuclear/cytosolic localization (Nuc/Cyt; middle panel) or an exclusive nuclear localization (Nuc; lower panel) were counted and expressed as percentage of ACOX1-Flag-expressing cells in the bar graph. Note that at least 200 cells were analysed per condition. Scale bar, 10 µm.

The *in vitro* import assays described above suggest that mACOX1 is a much better substrate for the PIM than dACOX1. An important question is whether this preference is maintained under *in vivo* conditions. To address this issue, we (co-)transfected COS-7 cells with plasmids encoding two epitope-tagged versions of ACOX1, one containing a functional PTS1 (2HA-ACOX1; see above) and the other lacking it (ACOX1-Flag). Next, we investigated whether the first protein can carry the second one to the peroxisome, and if so, with what efficiency. Control experiments (figure 4*a*), in which each of these plasmids was transfected alone, revealed a peroxisomal (panel I) and cytosolic (panel II) staining pattern for 2HA-ACOX1 and ACOX1-Flag, respectively. Interestingly, in a small number of cells (less than 5%), expression of ACOX1-Flag also resulted in a staining pattern that partially, but very weakly, overlapped with that of the peroxisomal marker protein PEX14 (figure 4*a*, panel III), suggesting that a minor amount of ACOX1-Flag was imported into peroxisomes piggybacked with endogenous ACOX1 (see below). In agreement with the peroxisomal localization observed for 2HA-ACOX1, western blot analyses of total cell extracts using an antibody directed to the 53 kDa polypeptide of ACOX1 revealed that the majority of this protein ran below the intact 74 kDa endogenous ACOX1 and immediately above its 53 kDa fragment, indicating that it was cleaved in the peroxisomal matrix (figure 4*b*). We next co-transfected cells with mixtures of the two plasmids, and determined the subcellular localization of each ACOX1 species by immunofluorescence at 1, 2 and 3 days post-transfection. To ensure that most ACOX1-Flag produced in these cells had the possibility to interact with newly synthesized 2HA-ACOX1, and thus to be imported into peroxisomes, a 1 : 10 mixture of the expression plasmids encoding ACOX1-Flag and 2HA-ACOX1, respectively, was used. Under these conditions, an exclusive peroxisomal localization was found for 2HA-ACOX1 regardless of the time point at which the immunofluorescence analyses were performed. By contrast, ACOX1-Flag displayed a cytosolic localization in more than 90% of the cells analysed at 1 day post-transfection (figure 4*c*, bar graph at the left-hand side). Interestingly, however, a small percentage of cells displayed a dual cytosolic and peroxisomal localization for ACOX1-Flag at this time point. The fraction of cells displaying such a distribution pattern increased over the next 2 days, but an exclusive peroxisomal localization for ACOX1-Flag could never be observed.

We were able to increase significantly the percentage of cells presenting a peroxisomal localization for ACOX1-Flag by transfecting cells with a 1 : 30 mixture of the plasmids encoding ACOX1-Flag and 2HA-ACOX1, respectively. As shown in figure 4*c* (bar graph at the right-hand side), an exclusive peroxisomal localization for ACOX1-Flag was found in approximately 5% of cells already at 1 day post-transfection. Similarly to the results above, the fraction of cells presenting this labelling pattern increased slowly during the two subsequent days. Together, these experiments strongly indicate that ACOX1-Flag can interact with 2HA-ACOX1 in the cytosol and use its PTS1 to reach the peroxisome. Thus, dimeric ACOX1 is also a substrate for the PIM. However, it is also evident from these data that targeting of ACOX1-Flag to the peroxisome, in contrast to that of 2HA-ACOX1, is a low-efficiency process occurring over a timescale of days.

The reason why ACOX1-Flag is poorly imported into the organelle could reflect difficulties of the Flag-tagged protein in interacting with 2HA-ACOX1. Although the *in vitro* oligomerization assay shown in figure 1*a* already suggests that this is not the case, an *in vivo* approach was used to test this possibility. Specifically, we co-expressed ACOX1-Flag with a 2HA-ACOX1 species containing three copies of a nuclear-localization signal (NLS) at its C-terminus (thus blocking its PTS1; see figure 4*a*, panel IV) in COS-7 cells and asked whether the nuclear targeted protein (2HA-ACOX1–3NLS) could transport ACOX1-Flag to the nucleus. Similarly to the experiment above, we used a 1 : 10 mixture of the plasmids encoding ACOX1-Flag and 2HA-ACOX1–3NLS, respectively. As shown in figure 4*d*, almost all ACOX1-Flag was found in the nucleus, thus suggesting that the Flag epitope does not interfere with the dimerization of ACOX1.

## 4. Discussion

The idea that most newly synthesized peroxisomal proteins are imported into the organelle after oligomerization in the cytosol has remained widely accepted during the last two decades. Besides all the studies referred to above that were used to develop and support this concept (see Introduction section), other arguments were frequently used to strengthen it. An important one, which is nowadays questionable [70,71], was that peroxisomes seemed to lack a protein-folding machinery [45,48,55]. Thus, newly synthesized peroxisomal matrix proteins should undergo folding and oligomerization in the cytosol, where such a machinery exists. Data on human alanine-glyoxylate amino transferase, a peroxisomal homo-dimeric enzyme, seemed to provide the proof-of-concept for this idea. Indeed, some mutations in the enzyme found in hyperoxaluria type-1 patients lead to the mistargeting of a fraction of the protein to the mitochondria. Since these mutations also affect dimerization of the enzyme, it was thus concluded that only the dimeric enzyme is competent for peroxisomal import (reviewed in [72,73]). However, an identical mistargeting phenomenon was recently described for human 2-enoyl-CoA hydratase/3-hydroxyacyl-CoA dehydrogenase (EHHADH), one of the few monomeric proteins of the peroxisomal matrix [74]. Indeed, studies in a family affected with inherited renal Fanconi's syndrome revealed that a single missense mutation near the N-terminus of this enzyme was sufficient to create a mitochondrial targeting sequence thus resulting in its mitochondrial mistargeting [75]. Clearly, there is no need to invoke a defect in the oligomerization of a peroxisomal matrix protein to explain its mistargeting to mitochondria (see also [76] for a discussion on this issue). Rather, the fact that a fraction of both mutant enzymes is missorted to mitochondria suggests that the mutations they harbour not only create mitochondrial targeting information but also interfere with their expedite folding, because only unfolded proteins are accepted by the mitochondrial import machinery [77].

The data presented in this work add to a number of observations suggesting that several newly synthesized peroxisomal matrix proteins arrive at the peroxisomal membrane still as monomers. A particularly interesting finding of our work is that both ACOX1 and UOX, similarly to catalase and sterol carrier protein x [23,61], can be easily obtained in a soluble, monomeric state. The solubility of all these proteins, together with the fact that their hydrodynamic properties are compatible with a globular conformation, suggests that they are already partially folded. With the exception of sterol carrier protein x, for which no evidence for *in vitro* homo-dimerization could be obtained thus far [23], monomeric ACOX1, UOX and catalase can all be converted into the corresponding oligomers *in vitro*. These findings suggest, on one hand, that these proteins are *bona fide* assembly intermediates, and, on the other hand, that (partial) folding and oligomerization of these monomeric proteins are not obligatory coupled events. Importantly, the data presented here show that the previously reported capacity of PEX5 to bind monomeric catalase, blocking its oligomerization [61], is also valid for ACOX1 and UOX, two proteins which together with catalase comprise one-third of the total matrix proteins found in rat/mouse liver peroxisomes [62,64].

Interestingly, *in vitro* import assays revealed that monomeric ACOX1 and UOX are more efficiently imported into peroxisomes than the corresponding oligomeric versions. The results of the co-transfection experiments presented in figure 4, showing that HA-ACOX1 acquires a peroxisomal localization in a much more efficient manner than ACOX1-Flag, are also compatible with this interpretation. While these findings suggest that the PIM displays a preference for monomeric proteins, they do not unveil the mechanistic reasons for such preference. Our *in vivo* data suggesting that import of dimeric ACOX1 is a low-efficiency process could be explained by simply assuming that interaction of monomeric ACOX1 with PEX5 is a much faster event than the dimerization of the enzyme in the cytosol. However, the *in vitro* import assays presented here suggest that the preference of the PIM for monomeric proteins may also have other reasons. For instance, it is possible that PEX5 binds monomeric proteins in a faster/stronger manner than it binds the corresponding oligomeric versions. Some data suggesting that this may be the case for catalase have been presented before [61]. Alternatively, the preference of the PIM for monomeric proteins might be exerted by the DTM itself. In this hypothetical scenario, the DTM would accept monomeric proteins having a near-native (more flexible) conformation more efficiently than already oligomerized (rigid) proteins. Discriminating between these possibilities will be a difficult task, requiring much more than the presently available qualitative data on the protein–protein interactions that govern the peroxisomal protein import pathway.

**Figure 5.**
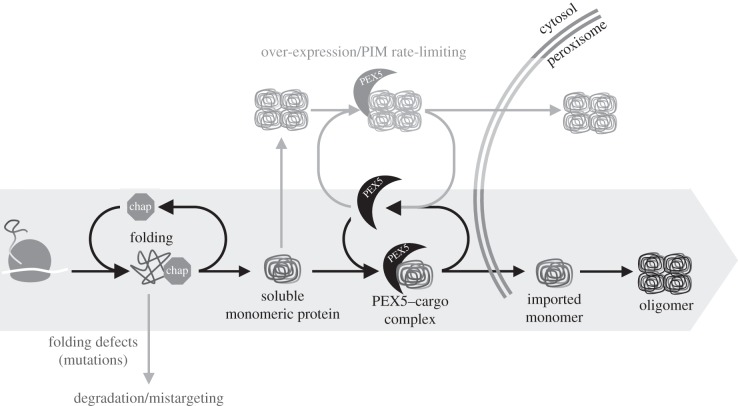
Working model for the initial steps of the peroxisomal matrix protein import pathway. Newly synthesized proteins are bound by chaperones during or immediately after their synthesis on cytosolic ribosomes. Successfully folded proteins are then released as near-native monomers and bound by cytosolic PEX5. These monomers are transported to the peroxisomal matrix where oligomerization can occur. Peroxisomal matrix proteins that do not fold correctly (e.g. as a result of mutation) are not released from the chaperones. Retained proteins may be degraded or mistargeted, as is the case of some mutant forms of alanine-glyoxylate amino transferase and 2-enoyl-CoA hydratase/3-hydroxyacyl-CoA dehydrogenase which target mitochondria [75,76]. Some proteins may also be imported after oligomerization. This pathway may be particularly used when matrix proteins are overexpressed and the import machinery becomes rate-limiting. Note that this pathway is rather inefficient for the two proteins characterized in this work and cannot be used for all those proteins that no longer expose their PTS upon oligomerization.

Although there is still much to be learned on how newly synthesized peroxisomal proteins are transported to the organelle matrix, the data presented here together with a number of previous findings (see Introduction section) support a model in which: (i) many newly synthesized peroxisomal proteins are folded by cytosolic chaperones and released as soluble monomers; (ii) these monomers are then bound by cytosolic PEX5, which blocks their oligomerization; and finally, (iii) these monomeric cargoes are translocated by a single PEX5 molecule into the matrix of the organelle where oligomerization occurs (figure 5).

## 5. Material and methods

### 5.1. Plasmids and recombinant proteins

The cDNAs encoding mouse ACOX1 (clone ID 5704873, Open Biosystems) and UOX (clone ID 5136328, Open Biosystems) were amplified by PCR using the primers 5′-GCTAATTCTAGAGCCACCATGAATCCCGATCTG-3′ and 5′-CGCCGTGGTACCTAGCATCAAAGCTTCGACTG-3′, and 5′-GCAGCATCTAGAGCCACCATGGCCCATTACC-3′ and 5′-CGCGCGGGTACCTTTCACAGCCTGGAAGGCA-3′, respectively, and cloned into a XbaI/KpnI digested pGEM4^®^ vector (Promega). To generate the plasmid pGEM4–2HA-UOX, primers 5′-AGCTTACCATGGGCTACCCCTATGATGTGCCCGATTACGCCTACCCATACGACGTCCCAGACTACGCTT-3′ and 5′-CTAGAAGCGTAGTCTGGGACGTCGTATGGGTAGGCGTAATCGGGCACATCATAGGGGTAGCCCATGGTA-3′, encoding two copies of the haemagglutinin (HA) tag, were annealed and cloned into a HindIII/XbaI digested pGEM4^®^ vector (Promega), originating pGEM4–2HA. Subsequently, the UOX cDNA amplified using the primers 5′-GCGCCGTCTAGAGCCCATTACCATGACAACT-3′ and 5′-CGCGCGGGTACCTTTCACAGCCTGGAAGGCA-3′ was inserted into the XbaI/KpnI sites of the pGEM4–2HA. To generate the pGEM4–2HA-ACOX1 plasmid (pMF1636), the cDNA encoding mouse ACOX1 was amplified by PCR (template: pGEM4–ACOX1; primers: 5′-GGAGGGTACCCATACGACGTCCCAGACTACGCTAATCCCGATCTGCGCAAG-3′ and 5′-GGGGAATTCAGCATCAAAGCTTCGACTGCAGGGGC-3′), digested with KpnI/EcoRI, and co-ligated with a HindIII/KpnI site-containing linker (prepared from the annealed oligonucleotides 5′-AGCTTACCATGGGCTACCCCTATGATGTGCCCGATTACGCCGGAGGGTAC-3′ and 5′-CCTCCGGCGTAATCGGGCACATCATAGGGGTAGCCCATGGTA-3′) into HindIII/EcoRI-restricted pGEM4. To generate the mammalian expression vector encoding 2HA-ACOX (pMF1808), the corresponding cDNA was amplified by PCR (template: pMF1636; primers: 5′-GGGGAATTCACCATGGGC TACCCCTATGATG-3′ and 5′-GGGGCGGCCGCTCAAAGCTTCGACTGCAGGGGC-3′), digested with EcoRI/NotI and ligated into EcoRI/NotI-restricted pEGFP-N1 (Clontech). To construct the mammalian expression vector encoding ACOX1-Flag (pMF1809), the cDNA encoding ACOX1 was amplified by PCR (template: pGEM4–ACOX1; primers: 5′-GGGGGCGGCCGCGCCACCATGGATCCCGATCTGCGCAAGGAG-3′ and 5′-GGGGAATTCAGCATCAAAGCTTCGACTGCAGGGGC-3′), digested with NotI/HindIII and ligated into pCMV-Tag4A (Stratagene). The mammalian expression vector encoding 2HA-ACOX1–3NLS (pOI16) was generated in two steps: first, a plasmid encoding myc-ACOX1–3NLS (pCL5) was constructed by ligating an EcoRI/BglII-restricted PCR fragment (template: pMF1636; primers: 5′-GGGAATTCGGATGGACCCCGATCTGCGCAAGG-3′ and 5′-GGAGATCTTCGACTGCAGGGGCTTCAAGTGC-3′) into EcoRI/BglII-restricted CL-Myc-DAO-3NLS (this plasmid was kindly provided by Dr Y. M. Go (Tuffs University, MA, USA)); next, the SalI/NotI fragment of pMF1808 was replaced by the SalI/NotI fragment of pCL5. To generate pGEM4–ACOX1-Flag, the corresponding cDNA was amplified by PCR (template: pMF1809; primers: 5′-GCTAATTCTAGAGCCACCATGAATCCCGATCTG-3′ and 5′-GCCGCGGGTACCAACTACTTATCGTCGTCATCC-3′) and subsequently cloned into the XbaI/KpnI-restricted pGEM4^®^ vector. The correctness of all plasmids was confirmed by DNA sequence analysis (LGC Genomics). The recombinant large isoform of mouse PEX5 [61], a protein comprising amino acid residues 315–639 of PEX5 (TPRs [78]), and TPRs containing the missense mutation N526K (TPRs (N526K), numbering of full-length PEX5 [79]) were obtained as previously described.

### 5.2. Synthesis of radiolabelled proteins

^35^S-labelled proteins were synthesized using the TnT T7 QuickCoupled transcription/translation kit (Promega) in the presence of ^35^S-methionine (specific activity > 1000 Ci mmol^−1^; PerkinElmer Life Sciences). Protein synthesis was allowed to proceed for the specified periods of time. Cycloheximide was used at 0.5 mM, final concentration. Chase incubations were performed at 30°C, as specified.

### 5.3. Immunoprecipitations

Radiolabelled proteins synthesized in the presence of 1 μM recombinant PEX5 were diluted to 500 µl with buffer A (50 mM Tris–HCl, pH 8.0, 150 mM NaCl, 1 mM EDTA–NaOH, pH 8.0, 10% (w/v) glycerol, 0.1% (w/v) Triton X-100) supplemented with 0.025% of bovine serum albumin (BSA) and 1 : 500 (v/v) mammalian protease inhibitor mixture (Sigma). The composition of translation product mixtures, prepared in the presence and absence of PEX5, was made identical by adding recombinant PEX5. Immunoprecipitation was done using 30 µl of anti-HA antibody agarose beads (Sigma) for 3 h at 4°C. Beads were washed four times with 150 µl of buffer A. Immunoprecipitated proteins were analysed by SDS-PAGE/autoradiography.

### 5.4. Sucrose gradients

Radiolabelled proteins were incubated for 5 min at room temperature in the presence or absence of 1 µM of either PEX5 or a control protein (soybean trypsin inhibitor), in 200 µl of buffer B (50 mM Tris–HCl, pH 7.5, 150 mM NaCl, 1 mM EDTA–NaOH, pH 8.0 and 1 mM DTT). The mixtures were then loaded onto the top of a continuous 5–30% (w/v) sucrose gradient in the same buffer (generated using a 107ip GRADIENT MASTER^TM^; BioComp, Canada) and centrifuged at 39 000 r.p.m. for 29 h at 4°C in a SW-41 Rotor (Beckman). Where indicated, 1 µM of either PEX5 or soybean trypsin inhibitor was included in the gradient solutions. Ovalbumin (3.6 S), BSA (4.3 S) and bovine immunoglobulins (6.9 S) were used as sedimentation coefficient standards. Fractionation of gradients, SDS-PAGE and autoradiography analyses were done as described [36]. Mouse liver peroxisomal matrix proteins were obtained by sonicating purified organelles (800 µg of protein) in buffer B supplemented with 1 : 500 (v/v) mammalian protease inhibitor mixture (Sigma) followed by centrifugation for 30 min at 100 000*g*. The supernatant was loaded onto the top of a sucrose gradient and centrifuged, as above.

### 5.5. *In vitro* import reactions

Rat liver PNS was prepared as described before [22]. *In vitro* import assays containing 500 µg of PNS and the radiolabelled protein were incubated for 45 min at 37°C, in 100 µl of import buffer (0.25 M sucrose, 20 mM MOPS-KOH, pH 7.4, 50 mM KCl, 3 mM MgCl_2_, 20 µM methionine, 2 μg ml^−1^ *N*-(trans-epoxysuccinyl)-l-leucine 4-guanidinobutylamide) containing 3 mM ATP, 2 mM glutathione, and recombinant PEX5 (1.5 nM for ACOX1 and 7.5 nM for UOX). Where indicated, TPRs or TPRs(N526K) (0.3 µM final concentration) was also added to assays. Note that incubation of radiolabelled ACOX1 and UOX with recombinant PEX5 before proceeding with the import assay, a strategy used before for sterol carrier protein x [23], resulted in only a modest increase in the import yields of ACOX1 and UOX (approx. 1.5-fold increase in 45 min import reactions). Thus, for practical reasons, this step was not included in the experiments described here. It is likely that the half-lives of the PEX5–ACOX1 and PEX5–UOX protein complexes are too short to benefit from this step, although further data are necessary to confirm this possibility. Protease treatment of import reactions was done using 400 µg ml^−1^ of proteinase K (final concentration) for 40 min, on ice. After protease inactivation with phenylmethylsulfonyl fluoride (500 µg ml^−1^, final concentration) for 2 min on ice, organelles were isolated by centrifugation and analysed as described before [28].

### 5.6. Cell culture, transfections and immunofluorescence microscopy

COS-7 cells (kindly provided by Dr M. Schrader (University of Exeter, UK)) were cultured at 37°C in a humidified 5% CO_2_ incubator in minimum essential medium Eagle *α* (Lonza) supplemented with 10% (v/v) fetal bovine serum superior (Biochrom AG), 2 mM Ultraglutamine-1 (Lonza) and 0.2% (v/v) Mycozap (Lonza). Cells were transfected using Invitrogen's Neon Transfection System (1050 V, 30 ms pulse width, 1 pulse). Samples for immunofluorescence microscopy were fixed and processed as described before [80]. The rabbit polyclonal antiserum against human PEX14 has been described elsewhere [81]. DAPI (Roche), the mouse anti-HA (Sigma) and anti-FLAG (Stratagene) antibodies, and the Alexa Fluor 488- (Invitrogen) or Texas Red- (Calbiochem) conjugated secondary antibodies were commercially obtained. Fluorescence was evaluated on a motorized inverted IX-81 microscope (Olympus) controlled by Cell-M software (Olympus). The technical specifications of the objectives, excitation and emission filters, and digital camera have been described elsewhere [82]. Fluorescence intensity versus distance plots (line scans) were generated using ImageJ software.

### 5.7. Miscellaneous

Isolation of highly pure peroxisomes from mouse liver by differential centrifugation and Nycodenz gradient purification was performed as described [83,84]. The antibodies directed to the 21 kDa and 53 kDa fragments of ACOX1 have been described before [85]. For the protease susceptibility assays, ^35^S-labelled proteins isolated from a sucrose gradient and native ACOX1 (from mouse liver purified peroxisomes) were diluted in import buffer and subjected to proteinase K digestion (10–400 µg ml^−1^, final concentration) in the presence or absence of 1% (w/v) Triton X-100, as specified, and incubated for 40 min on ice. After protease inactivation with phenylmethylsulfonyl fluoride, proteins were precipitated with trichloroacetic acid and processed as previously described [22].
